# Selected abstracts from the Breastfeeding and Feminism International Conference 2016: Chapel Hill, NC, USA, 20–22 March 2016

**DOI:** 10.1186/s13006-016-0082-9

**Published:** 2016-10-10

**Authors:** Lisa H. Amir, Alessandra Bazzano, Shelley Thibeau, Katherine P. Theall, Anna Blair, Karin Cadwell, Emily A. Bronson, Elizabeth C. Brooks, Jodine Chase, Ellen Chetwynd, Rebecca Costello, Kathryn Wouk, Lindsey Dermid-Gray, Stephanie Devane-Johnson, Cheryl Woods Giscombe, Miriam Labbok, Sally Dowling, Melanie Fraser, Jane Grassley, Deborah McCarter-Spaulding, Becky Spencer, Jennifer Hocking, Pranee Liamputtong, Sheree H. Keitt, Harumi Reis-Reilly, Miriam Labbok, Leslie Lytle, Mary Ann Merz, Kate Noon, Krista M. Olson, Ana M. Parrilla-Rodríguez, José J. Gorrín-Peralta, Melissa Pellicier, Zeleida M. Vázquez-Rivera, Melissa Pellicier, Jennifer L. Pemberton, Catherine McEvilly Pestl, Jennifer Pierre, Philip Noyes, Khushbu Srivastava, Sharon Marshall-Taylor, Jennifer Proto, Sarah Hyland, Laurie Brinks, Harumi Reis-Reilly, Martelle Esposito, Megan Phillippi, Cynthia L. Sears, Delores James, Cedric Harville, Kristina Carswell, Nicola Singletary, L. Suzanne Goodell, April Fogleman, Paige Hall Smith, Alison M. Stuebe, Amy G. Bryant, Anne Drapkin Lyerly, Cecilia Tomori, Amanda L. Watkins, Joan E. Dodgson, Jacqueline H. Wolf

**Affiliations:** 1 Judith Lumley Centre, La Trobe University, Melbourne, Australia; 2 Department of Global Community Health and Behavioral Sciences, Tulane University School of Public Health and Tropical Medicine, New Orleans, LA USA; 3 Ochsner Health System, New Orleans, LA USA; 4 Health and Wellness Program at Union Institute and University, Cincinnati, Ohio USA; 5 The Healthy Children Project, Inc, East Sandwich, MA USA; 6 University of South Florida, Tampa, Florida USA; 7 Lactation Consultant, Private Practice, Wyndmoor, PA USA; 8 News Analysis and Crisis Communications Consultant, Edmonton, Alberta Canada; 9 Carolina Global Breastfeeding Institute, Gillings Global School of Public Health, University of North Carolina at Chapel Hill, Chapel Hill, NC USA; 10 Women’s Birth & Wellness Center, Chapel Hill, NC USA; 11 Women, Infants and Children (WIC) Department, State of Nevada, Nevada, USA; 12 School of Nursing, University of North Carolina at Chapel Hill, Chapel Hill, NC USA; 13 Department of Maternal and Child Health, Carolina Global Breastfeeding Institute, Gillings School of Global Public Health, University of North Carolina at Chapel Hill, Chapel Hill, NC USA; 14 Department of Nursing and Midwifery, University of the West of England, Bristol, UK; 15 University of the West of England, Bristol, UK; 16 School of Nursing, Boise State University, Boise, ID USA; 17 Department of Nursing, Saint Anselm College, Manchester, NH USA; 18 College of Nursing, Texas Woman’s University, Denton, TX USA; 19 School of Science and Health, Western Sydney University, Richmond, Australia; 20 National Association of County and City Health Officials, Washington, DC USA; 21 Department of Maternal and Child Health, Carolina Global Breastfeeding Institute, UNC Gillings School of Global Public Health, University of North Carolina at Chapel Hill, Chapel Hill, NC USA; 22 Richmond Healthy Start Initiative, Richmond, VA USA; 23 St. Louis Breastfeeding Coalition, St. Louis, MO USA; 24 VA Alliance for Breastfeeding Laws, Richmond, VA USA; 25 Family Support Hawaii, Kealakekua, HI USA; 26 Maternal and Child Health Program, School of Public Health, University of Puerto Rico, San Juan, Puerto Rico; 27 Coalición para la Lactancia Materna en Puerto Rico, San Juan, Puerto Rico; 28 Coalición para la Lactancia Materna en Puerto Rico and Fundación Puertorriqueña para la Protección de la Maternidad y la Niñez, Promani, San Juan, Puerto Rico; 29 Behavioral Sciences, Community College of Baltimore County, Maryland, USA; 30 Lactation Consultant, Private Practice, Toronto, Canada; 31 Brooklyn District Public Health Office, Center for Health Equity, New York City Department of Health and Mental Hygiene, Brooklyn, NY USA; 32 Public Health Associate, Office for State, Tribal, Local and Territorial Support, Centers for Disease Control and Prevention, Atlanta, GA USA; 33 Ionia County Health Department, Ionia, MI USA; 34 National Association of County and City Health Officials, Washington, DC USA; 35 National WIC Association, Washington, DC USA; 36 Association of Maternal & Child Health Programs, Washington, DC USA; 37 University of Florida, Gainesville, FL USA; 38 UF Health-Shands Hospital, Gainesville, FL USA; 39 Department of Food, Bioprocessing and Nutrition Sciences, North Carolina State University, Raleigh, NC USA; 40 Public Health Education and Center for Women’s Health and Wellness, University of North Carolina at Greensboro, Greensboro, NC USA; 41 Division of Maternal-Fetal Medicine, University of North Carolina School of Medicine, Chapel Hill, NC USA; 42 Department of Maternal and Child Health, Gillings School of Global Public Health, University of North Carolina at Chapel Hill, Chapel Hill, NC USA; 43 Division of Family Planning, Dept of Ob/Gyn, UNC School of Medicine, University of North Carolina at Chapel Hill, Chapel Hill, NC USA; 44 Department of Social Medicine, UNC School of Medicine, and the Center for Bioethics, University of North Carolina at Chapel Hill, Chapel Hill, NC USA; 45 Johns Hopkins Bloomberg School of Public Health, Baltimore, MD USA; 46 Global Lactation Education Associates, Raleigh, NC USA; 47 Maternal Child Health Nursing Research, St. Louis University, St. Louis, MO USA; 48 Department of Social Medicine, Ohio University Athens, Ohio, USA

## Abstract

A1. Infant feeding and poverty: a public health perspective in a global context

Lisa H. Amir

A2. Mothers’ experiences with galactagogues for lactation: an exploratory cross sectional study

Alessandra Bazzano, Shelley Thibeau, Katherine P. Theall

A3. The motherhood journey and breastfeeding: from self-efficacy to resilience and social stigma

Anna Blair, Karin Cadwell

A4. Breastfeeding as an evolutionary adaptive behavior

Emily A. Bronson

A5. Conflict-of-interest in public health policy: as real as that logo on your website

Elizabeth C. Brooks

A6. Co-opting sisterhood and motherhood: behind the scenes of Similac’s aggressive social media campaigns

Jodine Chase

A7. The exclusion of women from the definition of exclusive breastfeeding

Ellen Chetwynd, Rebecca Costello, Kathryn Wouk

A8. Healthy maternity policies in the workplace: a state health department’s experience with the “Bring Your Infant to Work” program

Lindsey Dermid-Gray

A9. Implications for a paradigm shift: factors related to breastfeeding among African American women

Stephanie Devane-Johnson, Cheryl Woods Giscombe, Miriam Labbok

A10. Social experiences of breastfeeding: building bridges between research and policy: an ESRC-funded seminar series in the UK

Sally Dowling

A11. Manager’s perspectives of lactation breaks

Melanie Fraser

A12. The challenging second night: a dialogue from two perspectives

Jane Grassley, Deborah McCarter-Spaulding, Becky Spencer

A13. The role of lactation consultants in two council breastfeeding services in Melbourne, Australia – some preliminary impressions

Jennifer Hocking, Pranee Liamputtong

A14. Integrating social marketing and community engagement concepts in community breastfeeding programs

Sheree H. Keitt, Harumi Reis-Reilly

A15. What happens before and after the maternity stay? Creating a community-wide Ten Steps approach

Miriam Labbok

A16. #RVABREASTFEEDS: cultivating a breastfeeding-friendly community

Leslie Lytle

A17. Public health vs. free trade: a longitudinal analysis of a global policy to protect breastfeeding

Mary Ann Merz

A18. Legislative advocacy and grassroots organizing for improved breastfeeding laws in Virginia

Kate Noon

A19. Breastfeeding and the rights of incarcerated women

Krista M Olson

A20. Barriers and support for Puerto Rican breastfeeding working mothers

Ana M. Parrilla-Rodríguez, José J. Gorrín-Peralta Melissa Pellicier, Zeleida M. Vázquez-Rivera

A21. Pumping at work: a daily struggle for Puerto Rican breastfeeding mothers in spite of the law

Melissa Pellicier

A22. “I saw a wrong and I wanted to stand up for what I thought was right:” a narrative study on becoming a breastfeeding activist

Jennifer L. Pemberton

A23. Peer breastfeeding support: advocacy and action

Catherine McEvilly Pestl

A24. Good intentions: a study of breastfeeding intention and postpartum realities among first-time Central Brooklyn mothers

Jennifer Pierre, Philip Noyes, Khushbu Srivastava, Sharon Marshall-Taylor

A25. Women describing the infant feeding choice: the impact of the WIC breastfeeding classes on infant feeding practices in Ionia, Michigan

Jennifer Proto, Sarah Hyland Laurie Brinks

A26. Local and state programs and national partnership to reduce disparities through community breastfeeding support

Harumi Reis-Reilly, Martelle Esposito, Megan Phillippi

A27. Beyond black breastfeeding week: instagram image content analysis for #blackwomendobreastfeed/#bwdbf

Cynthia L. Sears, Delores James, Cedric Harville, Kristina Carswell

A28. Stakeholder views of breastfeeding education in the K-12 environment: a review of the literature

Nicola Singletary, L. Suzanne Goodell, April Fogleman

A29. “The Breastfeeding Transition”: a framework for explaining changes in global breastfeeding rates as related to large-scale forces shaping the status of women

Paige Hall Smith

A30. Breastfeeding, contraception, and ethics, oh my! Advocacy and informed decision-making in the post-partum period

Alison M. Stuebe, Amy G. Bryant, Anne Drapkin Lyerly

A31. A hard day’s night: juggling nighttime breastfeeding, sleep, and work

Cecilia Tomori

A32. Empowering change in Indian country through breastfeeding education

Amanda L. Watkins, Joan E. Dodgson

A33. Servants and “Little Mothers” take charge: work, class, and breastfeeding rates in the early 20^th^-century U.S.

Jacqueline H. Wolf

## A1. Infant feeding and poverty: a public health perspective in a global context

### Lisa H. Amir (L.Amir@latrobe.edu.au)

#### Judith Lumley Centre, La Trobe University, Melbourne, Australia

**Background**

Breastfeeding rates vary widely around the world, and change over time. Currently, as summarised in the recent *Lancet* series, poorer women in low and middle-income countries have higher rates of breastfeeding than richer women. In contrast, in high-income countries infants in poorer families are less likely to be breastfeed than infants in more advantaged families. Evidence from Australia and Canada suggests that this gap between rich and poor in developed countries is widening and contributes to the health inequities faced by families living in poverty. Children growing up in poverty have poorer health and development. The human brain undergoes huge growth in the early years of life. Brain scanning techniques show that the increase in total grey matter volume is significantly higher in children from wealthy families compared to poor children. The imaging differences are also reflected in better cognitive development in the children from higher socioeconomic status families. New research suggests that breastfeeding may reduce this income-related difference.

**Discussion**

Food insecurity can be defined as ‘limited or uncertain availability of nutritionally adequate and safe foods or the limited or uncertain ability to acquire such foods in socially acceptable ways’. This is a common situation for people experiencing disadvantage and leads to worse health status. Libby Salmon discusses the concepts of food security as they apply to infants and young children:

- food appropriateness
- availability
- accessibility
- affordability
- utilization and
- stability of supply.

Salmon explains that high rates of optimal breastfeeding are required for this group to be food secure, and suggests that “Food security provides an analytical **framework and overarching policy imperative** that may help international agencies, governments and community organizations to better address conflicts between health, agriculture and trade, all of which contribute to low breastfeeding rates and unregulated trade and marketing of breast milk substitutes. Existing policies fail to account for human rights and the unpaid work of breastfeeding women” [1]. Public health focuses on populations rather than individuals. The underlying concept is that changing a risk factor across a whole population by just a small amount can have a great impact on the community. Health promotion is a social and political process, aiming to strengthen individual skill and capabilities; to change social, environmental, economic conditions, to improve and maintain health. We can use these health promotion steps to address inequities in breastfeeding:

1. A system for intelligence gathering
2. Clear policy, legislation and regulation
3. Communication of information
4. Provision of primary services
5. Sharing of responsibility across sectors
6. Involvement of communities

**Conclusion**

“As individuals, women are powerless to counter the complexity of societal forces that interfere with exclusive breastfeeding their infants for six months. What is required are ‘structural changes . . . to society that will enable all mothers to breastfeed with assurance and safety’ [2].

**References**

1. Salmon L: **Food security for infants and young children: an opportunity for breastfeeding policy?** *Int Breastfeed J* 2015, **10**:7.

2. Beasley A, Amir LH: **Infant feeding, poverty and human development**. *Int Breastfeed J* 2007, **2**:14.

## A2. Mothers’ experiences with galactagogues for lactation: an exploratory cross sectional study

### Alessandra Bazzano^1^, Shelley Thibeau^2^, Katherine P. Theall^1^

#### ^1^Tulane University School of Public Health and Tropical Medicine, Department of Global Community Health and Behavioral Sciences, New Orleans, LA, USA; ^2^Ochsner Health System, New Orleans, LA, USA

##### **Correspondence:** Alessandra Bazzano (abazzano@tulane.edu) – Tulane University School of Public Health and Tropical Medicine, Department of Global Community Health and Behavioral Sciences, New Orleans, LA, USA

**Background**

Important progress has been made in the last decade to improve breastfeeding initiation and duration using the Ten Steps to Successful Breastfeeding guidelines recommended by the World Health Organization and UNICEF. In the US, breastfeeding rates across the country have steadily improved. Despite this progress, there remain barriers, and additionally, a consumer interest in the use of galactagogues to augment or improve milk production. Herbal galactagogues (such as fenugreek, fennel, blessed thistle, goats rhue and others) are widely available through drug stores, health food stores, and online. Marketing data indicate that many lactating women purchase and utilize these products, yet information on their safety and efficacy is lacking. Likewise, there is limited research on the use of pharmaceutical galactagogues, which may cause side effects and are typically prescribed “off label” for lactation.

**Methods**

To better understand mothers’ experiences with pharmaceutical and herbal galactagogues, we undertook an online survey using a convenience sample of 188 mothers. The survey consisted of 11 demographic and 17 items exploring the mothers’ experiences. The study population consisted of all respondents who had experienced lactation whether or not they gave birth. Additionally, we explored sources of information on galactagogues, and likelihood of recommending their use in the future. Distribution of the survey link with invitation to participate utilized the following methods: contacts within community breastfeeding support programs, snowball sampling, and social media. Mothers were informed that their participation was voluntary, no identifiable information would be collected, and that participation would not be remunerated, but could guide future research on galactagogue use among lactating women. Survey distribution occurred from 1 November 2015 to 1 April 2016. The study was reviewed by both the Ochsner Health System and Tulane University Institutional Review Boards.

**Results**

Analyses to date have produced the following results: the majority of respondents were White, breastfed one child, employed, and college educated. Twenty-seven states were represented; with the majority of responses from Louisiana (42 %) and Kansas (10 %). More than 70 % of respondents had ever felt that they were not making enough milk while breastfeeding and less than half consulted about a provider about this problem (typically a lactation counselor). One third of respondents had supplemented with breast milk substitutes. The most likely reported source of information regarding supportive aids to increase milk production was the Internet. While the majority of mothers used a breast pump or manual expression to improve milk production (64 %), a large proportion also used fenugreek and milk thistle. Nearly half of mothers had heard of pharmaceutical galactagogues, domperidone or metoclopramide, but a very small percentage (4 %) had used either. The importance of provider recommendation was not applicable for the majority of respondents.

**Conclusion**

Gathering information on mothers’ experiences of and perceptions about galactagogues may expand our knowledge for better lactation support. As there is limited scientific evidence on the safety and efficacy of galactagogues, understanding mothers’ experiences is crucial to advocating for women, and supporting their breastfeeding goals, while protecting their health and that of their infant.

## A3. The motherhood journey and breastfeeding: from self-efficacy to resilience and social stigma

### Anna Blair^1,2^, Karin Cadwell^1^

#### ^1^Health and Wellness Program at Union Institute and University, Cincinnati, Ohio, USA; ^2^The Healthy Children Project, Inc., East Sandwich, MA, USA

##### **Correspondence:** Anna Blair (Anna@healthychildren.cc) – Health and Wellness Program at Union Institute and University, Cincinnati, Ohio, USA

**Introduction**

Although almost universally acknowledged as behavior that potentially improves public health outcomes and decreases health care costs, many women find breastfeeding challenging and fraught with obstacles. Mothers experience social stigmas, a feeling that others disapprove of them in some way, related to choices they have made during their motherhood journey. A considerable number of studies have examined self-efficacy in relation to breastfeeding mothers: the strength of their belief that they can reach their infant feeding goals. Resilience and stigma have not been as widely investigated. It is important to consider how lactation care providers can best support a woman’s resilience when she faces breastfeeding challenges and how to reduce stigmas around infant feeding.

**Perspective**

Some women handle obstacles and grief and withstand the stress while others struggle more. Catastrophes and tragedies are a life expectation and can happen even during the joy of the perinatal event. Helping a mother to grow her capacity for resilience during pregnancy and lactation can carry over into other aspects of her life. Resilience is a learned trait. If care providers include purposeful resilience building (and an awareness of the negative power of stigmas) in breastfeeding interactions, rather than focusing on the mother’s goals, breastfeeding outcomes may be positively affected. Our work suggested that health care providers who support breastfeeding mothers should be aware of:

- The difference between self-efficacy and resilience
- the complexity and negative power of stigmas
- how mothers experience stigmas during their perinatal journey
- possible factors that contribute to resilience with participate emphasis on pregnancy and lactation
- how to help women develop resilience as they experience breastfeeding challenges
- how to help women experiencing stigmas

## A4. Breastfeeding as an evolutionary adaptive behavior

### Emily A. Bronson (em.bronson@gmail.com)

#### University of South Florida, Tampa, Florida, USA

**Background**

Solitary infant sleep arose from the medicalization of childbirth and parenting. Current societal norms and medical institution recommendations include blanket statements against co-sleeping to prevent suffocation and sudden infant death syndrome (SIDS). These recommendations often do not differentiate between sleeping on a bed or a couch, whether the mother is breastfeeding or formula feeding, or whether the mother is of high or low socioeconomic status. As evidenced by our evolutionary past, studies of primates, and current research on breastfeeding and infant sleep, breastfeeding and co-sleeping go hand-in-hand. Disruption of sustained mother-infant contact, such as solitary sleeping, is a hindrance to breastfeeding, causing a decrease in milk production, and less likelihood of breastfeeding throughout the night and beyond 3 months. In addition to promoting lactation, bed-sharing also compensates for the infant’s immature neurological, immunological, and developmental altricial status. Recent research suggests that both breastfeeding and bed-sharing may also be closely associated with the prevention of sudden infant death syndrome (SIDS). Particularly, there are crucial differences in how breastfeeding and bottle-feeding moms interact physiologically with their bed-sharing infants. Co-sleeping is more common than expected; breastfeeding often leads mothers who did not intend to bed-share to do so at least part of the night, sometimes doing so unsafely on sofas or under other unsafe circumstances.

**Discussion**

As an increasing number of parents are encouraged to breastfeed, the number of infants who bed-share will also increase. Thus, parents need to be provided with the information to make informed decisions using information on safe co-sleeping and based on their own individual circumstances. Anthropologists James McKenna and Lee Gettler proposed a brand new term “breastsleeping” to more accurately refer to breastfeeding-bed-sharing mother-infant pairs [1]. When considering public health recommendations aimed at reducing the risk of SIDS, health professionals need to take into account the vastly different context of breastfeeding and non-breastfeeding dyads. A lack of information and restrictive policies toward infant sleep undermines parents’ right to make informed parenting choices. Messages stating that all bed-sharing is harmful are detrimental to maternal-infant health in ways that we are just beginning to understand, and shame parents who do safety breastsleep. Discouraging breastsleeping is also disproportionately detrimental to impoverished mothers who often return to work soon after giving birth, causing further disruption of lactation. Breastsleeping may be an evolutionary adaptive behavior that plays a key role in our health, and discouraging bed- sharing may actually be operating against our evolved biology.

**References**

1. McKenna J, Gettler LT. There is no such thing as infant sleep, there is no such thing as breastfeeding, there is only breastsleeping. Acta Paediatr*.* 2015: 105(1): 17-21

## A5. Conflict-of-interest in public health policy: as real as that logo on your website

### Elizabeth C. Brooks (liz.brooks@yahoo.com)

#### Lactation Consultant, Private Practice, Wyndmoor, PA, USA

**Introduction**

Healthcare providers (HCPs) are required by the ethics of their professions to avoid a conflict-of-interest in their clinical work. Professional behaviors are meant to be motivated by providing good health care, free of overt or implied influence from profit-motivated manufacturers and marketers of pharmaceuticals, medical devices, or any of the products covered by the *International Code of Marketing of Breast-milk Substitutes.* The HCP’s duty of care is to the patient/client, whose well-informed decision about care is made after an evidence-based risk-and-benefits discussion with the HCP. Similarly, policy and decision-makers in public health owe a duty of care to the population-at-large, constructing rules and obligations that provide the greatest benefit, at the lowest risk, for the greatest number of people within the geopolitical region. There is increasing financial pressure in 2016 for public health officials to “collaborate” with any-and-all parties claiming a stake in the public’s good health. Major food- and infant-formula-producing companies are partnered with international public health, dietetic and pediatric associations in healthy eating and anti-obesity campaigns. Examples include: Mead-Johnson partnering with the American Academy of Pediatrics on childhood nutrition and obesity campaigns, and funding an Alberta Health Services educational day about NICU infants; Nestle funding the 2016 Lebanese Pediatric Society conference; both the Association of Women’s Health, Obstetrics and Neonatal Nurses (AWHONN) and the Academy of Nutrition and Dietetics (AND) offering special notice and consideration to prime corporate sponsors on their websites.

**Perspective**

Corporations are also funding education for HCPs, including use of branded- materials and those with logos. The motives for participation by commercial interests are narrower than the motives (and obligations) of public health officials, whose duty of care is to establish baseline recommendations for public health policy. This session explored whether public health and corporate interests can “come to the same table” to design policies uninfluenced by commercial motives.

**Conclusion**

Commercial interests cannot ignore their duty to shareholders/owners to seek profit, despite announced altruistic or public health motives. Healthcare providers and their professional associations should not accept financial inducements and gifts from commercial interests, as doing so unwittingly affects impartiality.

## A6. Co-opting sisterhood and motherhood: behind the scenes of Similac’s aggressive social media campaigns

### Jodine Chase (chase.jodinechase@gmail.com)

#### News Analysis and Crisis Communications Consultant, Edmonton, Alberta, Canada

**Background**

Similac maker Abbott’s sophisticated direct-to-consumer campaign harnesses the power of emotional appeal. Its StrongMom campaign signs women up by the thousands to a “membership rewards” program promising hundreds of dollars’ worth of coupons, weekly emails offering cheerful pregnancy tips and “expert” nutrition advice, and trendy gifts like a free Shutterfly photobook or a stylish messenger bag. A social media campaign drives mothers-to-be to the Similac website - “StrongMoms sign up here.” Women give up their addresses so the rewards of membership, which includes many free ounces of infant formula, can be shipped directly to their homes. The campaign is reinforced by encouraging YouTube videos designed to go viral, propelled by social media hashtag-slogans like #sisterhoodunite and #endmommywars.

**Campaign tools and strategy**

The first YouTube video was a faux parody, “The Mother ‘Hood’”, telling women it’s time to stop shaming those who don’t live up to parenting ideals, because the “mommy wars” are dividing the sisterhood of mothers who all have one common goal - a safe and healthy baby. Next up was a longer “crockumentary,” a fake documentary launched with enough hype to rival a blockbuster Hollywood film release. The videos make good use of the marketing strategy of emotional appeal.

**Conclusion**

What’s driving this very successful campaign? This paper used Abbott’s own marketing and creative staff explanations of their campaign goals to expose Abbott’s real brand positioning strategy - to make Similac be the only brand that truly understands how a breastfeeding mom really feels, while isolating her from genuine expert and peer breastfeeding support. Abbott erodes faith in lactation consultations and the real sisterhood, our very effective breastfeeding peer support groups. With no timely and effective professional or peer support, Abbott then steps in with its 24-7 nutritional advice hotlines and formula samples from the brand that mom recalls made her feel good.

## A7. The exclusion of women from the definition of exclusive breastfeeding

### Ellen Chetwynd^1,2^, Rebecca Costello^2^, Kathryn Wouk^1^

#### ^1^Carolina Global Breastfeeding Institute, Gillings Global School of Public Health, University of North Carolina at Chapel Hill, Chapel Hill, NC, USA; ^2^Women’s Birth & Wellness Center, Chapel Hill, NC, USA

##### **Correspondence:** Ellen Chetwynd (chetwynd@email.unc.edu) – Carolina Global Breastfeeding Institute, Gillings Global School of Public Health, University of North Carolina at Chapel Hill, Chapel Hill, NC, USA

**Background**

Exclusive breastfeeding is defined as an infant who “has received only breastmilk from his or her mother or a wet nurse, or expressed breast milk, and no other liquids or solids with the exception of drops or syrups consisting of vitamins, mineral supplements, or medicines” [1]. Within this definition of exclusive breastfeeding are vastly different maternal experiences. We used a dataset of women from an out of hospital birth center who have high intention and high exclusivity to define variations of lived breastfeeding experiences within the research label of ‘exclusive breastfeeding’.

**Methods**

We conducted two surveys of birth center patients: the first completed at 3 months postpartum and the second completed at any time during the first 16 months postpartum. Both surveys asked women how their babies were fed at different time points during the first 6 months, with response options of “Nursed at breast,” “Mom’s pumped milk,” “Formula,” “Donor milk,” and/or “Solid foods.” Data from both surveys were combined to present trends in the following methods of infant feeding: 1) exclusive human milk feeding, defined as at-breast, pumped, or donor milk; 2) exclusive mother’s own milk feeding, defined as at-breast or pumped milk; 3) exclusive at-breast feeding; 4) exclusive pumped milk feeding, and 5) any breastfeeding across the first 6 months postpartum.

**Results**

Our analytic sample includes 206 mothers from the 3-month survey and 240 from the 16-month study for a total of 446 respondents. In the first week after birth, 96 % of mothers were able to exclusively human milk feed, dropping slightly to 93 % at 1 month, and increasing again to 96 % at 2 months before declining at 3 and 6 months. 84 % exclusively fed mother’s own milk (whether at-breast or pumped) in the first week, increasing to 89 % at 1 month, 93 % at 2 months, and 91 % at 3 months before declining to 61 % at 6 months. Approximately 78 % of mothers provided only at-breast milk in the first week, declining to 35 % at 2 months, and dropping to 25 % by 6 months. No respondents exclusively pumped milk in the first week postpartum, but 1 % exclusively pumped at 1 month and 3 % at 2 and 3 months. All respondents gave breastmilk in the first week postpartum, and 97 % were continuing to give some amount of breastmilk at 6 months postpartum.

**Discussion**

We contrast definitions of exclusive breastfeeding as ‘at the breast’, ‘own mother’s milk’, and ‘human milk’ exclusive breastfeeding. We draw attention to a subset of women within this well-supported cohort who move between definitions, for example those who initially supplemented with human milk but were able to attain exclusive mothers own milk feeding in the first month of life. Mothers may apply the definition of “exclusive breastfeeder” differently to themselves. Researchers need to be aware of these differences as they frame their research questions.

**References**

1. World Health Organization. Indicators for Assessing Breastfeeding Practices: Reprinted Report from an Informal Meeting. Geneva: Division of Child Health and Development, WHO/CDD/SER/91.14. 1991

## A8. Healthy maternity policies in the workplace: a state health department’s experience with the “Bring Your Infant to Work” program

### Lindsey Dermid-Gray (lgray@health.nv.gov)

#### Women, Infants and Children (WIC) Department, State of Nevada, Nevada, USA

**Background**

Forty-five percent of the workforce in the United States is made up of women who make an invaluable contribution to their workplace. Unfortunately, 22 percent of women don’t return to work after their first baby is born. The Bring Your Infant to Work program addresses the current workplace/workforce mismatch by allowing for an easier transition back into work for the mother and a much greater likelihood of success in meeting breastfeeding goals. The application of this policy can be tailored to meet a variety of working environments around the world.

**Method**

Qualitative and quantitative surveys were sent out to all the mothers (n = 24) and coworkers of mother/baby dyads (n = 424) who participated in this program at Nevada's Department of Health and Human Services between 2009 and 2013.

**Results**

Participating mothers reported an average maternity leave of eight weeks, average amount of savings on day care of $2,850.00, an average duration of breastfeeding of 12.5 months, and an average number of sick days taken due to infant illness of two. Of the coworkers who had experienced direct proximity to an infant in the program, 82 % reported having a positive experience and 83 % reported a favorable view of the policy.

**Conclusions**

The Bring Your Infant to Work program is one of the most progressive healthy maternity policies to date. When implemented correctly, it can lead to numerous benefits for families and businesses, including: reduced maternity leave, an easier transition back into to the workplace, increased meeting and exceeding of breastfeeding goals, continued access to income and health and retirement benefits, and the maintenance of a large and diverse workforce for employers.

## A9. Implications for a paradigm shift: factors related to breastfeeding among African American women

### Stephanie Devane-Johnson^1^, Cheryl Woods Giscombe^1^, Miriam Labbok^2^^

#### ^1^School of Nursing, University of North Carolina at Chapel Hill, Chapel Hill, NC, USA; ^2^Carolina Global Breastfeeding Institute, Department of Maternal and Child Health, Gillings School of Global Public Health, University of North Carolina at Chapel Hill, Chapel Hill, NC, USA

##### **Correspondence:** Stephanie Devane-Johnson (sdevane@email.unc.edu) – School of Nursing, University of North Carolina at Chapel Hill, Chapel Hill, NC, USA

^^^Deceased

**Background**

Breastfeeding has widespread health benefits for infants, mothers and society. However, not all populations of women engage in breastfeeding at high rates. Data from the National Immunization Survey indicates that 66 % of African American mothers initiate breastfeeding but only 39 % continue to breastfeed at 6 months. In contrast, 84 % of White mothers initiate breastfeeding and 45 % are still breastfeeding 6 months; for Hispanics, 83 % initiate and 46 % continue to 6 months [1]*.* Although efforts have been made to address the disproportionately low rates of breastfeeding among African American women, disparities continue to exist. A potentially under explored area of interest may be socio-historical factors unique to African Americans.

**Objective**

This study sought to identify and gain a better understanding of socio-historical factors that influence breastfeeding beliefs and behaviors among contemporary African American women. Socio-historical factors include events, experiences and other phenomena that have been socially, generationally and culturally passed down and integrated into families and communities that influence health beliefs and health behaviors.

**Methods**

Six focus groups (three breastfeeding and three formula feeding), were conducted May 2015- September 2015. African American women who had given birth to at least one full term infant were purposively sampled, and stratified by age (18-29, 30-50 and 51+). The goal of the focus groups were to: 1) describe African American mothers thought processes in making infant feeding decisions; 2) describe cultural factors influencing African American mothers perceptions of infant feeding decisions and 3) identify possible connections between the socio-historical factors of African American women’s collective experiences and contemporary breastfeeding decisions.

**Results**

The following themes were identified: 1) negative feeling about breasts; 2) knowledge deficit/misinformation about breastfeeding; 3) breast as sexual not functional; 4) negative historical influences (i.e. mammy, wet-nursing, aggressive formula marketing) and 5) infant feeding traditions within families. These results support the use of socio-historical frameworks for guiding practice and research, including embodiment, historical trauma and the PEN-3 model developed by Collins Airhihenbuwa. The unique aspect of the PEN-3 model is that it addresses health disparities and health promotion from a three-domain approach. Each domain has three dimensions, accounting for the acronym ‘PEN’. First, cultural identity includes: A) Person; B) Extended family, and C) Neighborhood. Secondly, relationships and expectations include: A) Perceptions, B) Enablers, and C) Nurtures. The third domain is cultural empowerment and it includes factors that are: A) Positive, B) Existential (unique), and C) Negative.

**Conclusion:** Findings from this study can be used to develop new evidenced-based, culturally sensitive interventions to enhance breastfeeding in the African American community.

**References**

1. National Immunization Survey (2016). Rates of Any and Exclusive Breastfeeding by Socio-demographics among Children Born in 2013. Retrieved August 9, 2016 from https://www.cdc.gov/breastfeeding/data/nis_data/rates-any-exclusive-bf-socio-dem-2013.htm.

## A10. Social experiences of breastfeeding: building bridges between research and policy: an ESRC-funded seminar series in the UK

### Sally Dowling (sally.dowling@uwe.ac.uk)

#### Department of Nursing and Midwifery, University of the West of England, Bristol, UK

**Background**

Dr Sally Dowling (UWE), Dr Kate Boyer (Cardiff University), Dr Julie Mytton (UWE) and Prof David Pontin (University of South Wales) received funding from one of the major UK research funding councils, the ESRC (Economic and Social Research Council) to run series of ‘seminars’ (day conferences) in 2015 and 2016. Under the title ‘Social experiences of breastfeeding: building bridges between research and policy’ the six seminars in the series bring together academics from a range of disciplines with those involved in breastfeeding education, practice, activism, and policy. Our aim is to further understanding of women’s embodied, affective and day-to-day practices in trying to breastfeed through talking about these experiences across a series of events.

**Progress**

As of BFIC 2016, four events had been held in Bristol and Cardiff, with two more planned for 2016. Each was well-attended by academics, local and national policy makers, a range of practitioners (both professional and voluntary), Early Career Researchers/PhD students and midwifery and public health students. Attendees include a funded ‘core group’; this enables the continuation of discussions across and between seminars. Presentations on each occasion have been from one international academic and two UK academics, plus one person from policy and practice. Around 40 people attended each event and all events have been well evaluated. The following statements are from the evaluations:

- ‘It’s a great seminar series. One thing I particularly like is the idea of getting practitioners and academics together – such a wonderful opportunity!’ (Early Career Researcher)
- ‘. . . The seminar caused me to challenge some of my current practice and therefore change mine and colleagues current practice with regard to young mothers’. (Breastfeeding Nurse Advisor, NICU)
- ‘The March seminar was particularly interesting from a practice point of view with the presentations on shame and longer term breastfeeding. I shall be sending the podcast links to all breastfeeding counsellors with a recommendation to listen.’ (BF counsellor)

**Outcomes**

Planned outcomes include an edited book but already other outcomes are apparent, including a number of developing collaborations for research bidding and paper writing. As a direct consequence of this work Dowling and Boyer have attended the All Party Parliamentary Group on Infant Feeding and Inequalities; this involvement is on-going. Ways to capture emerging links between academics, policy-makers and practitioners and opportunities for influencing policy are being explored as the series progresses. This presentation outlined the rationale for applying for the seminar series funding and discussed the progress of the project to date. Drawing on data about attendees and from evaluations the discussion reflected on the process and on the success, to date, of ‘building bridges between research and policy’.

## A11. Manager’s perspectives of lactation breaks

### Melanie Fraser (melanieafraser@gmail.com)

#### University of the West of England, Bristol, UK

**Background**

There is a mismatch between health recommendations to breastfeed babies for up to 2 years or beyond, and UK employment law provisions, in which maternity leave is commonly up to one year with maternity employment protections normally ceasing after return to work.

The study explored the perspectives and views of employers and managers concerning the context for lactation breaks in one public sector organisation, access to relevant policies, the perceived legal position and views of managers around sustaining lactation on return to work. The research questions were designed to identify: (1) issues triggered for managers by employees combining breastfeeding or lactation and employment; (2) how managers understand and access the law concerning lactation breaks; and (3) views managers express with regard to the different ways in which a mother may sustain lactation on her return to work.

**Methods**

This qualitative study utilised snowballing sampling strategy to access and recruit participants. Interviews were conducted with twenty-seven managers and key personnel of a large family-friendly organisation in 2013, selected for the deviant level of support for lactation breaks. Documentary analysis was also utilised. Interviews were audio recorded and transcribed verbatim. Inductive thematic analysis was applied using NVIVO to discern themes.

**Results**

There are some gaps in provision for lactation breaks and potential barriers for staff contemplating them. Participants described support and concerns, demonstrating conflicted attitudes. Themes include support for combining lactation and employment; concerns about lactation; following organisational policy; questioning social policy and reservations about communication. There is some degree of contradiction between these over-arching themes. There was limited call for law reform and the topic was perceived as primarily a human resources issue. All forms of lactation break were associated with ambiguous attitudes and reservations.

**Implications**

Despite a high level of support for the concept of lactation breaks among managers there were concerns over potentially problematic issues. Breastfeeding at work triggers a workplace risk assessment rather than consideration of the potential risks of stopping breastfeeding early.

## A12. The challenging second night: a dialogue from two perspectives

### Jane Grassley^1^, Deborah McCarter-Spaulding^2^, Becky Spencer^3^

#### ^1^School of Nursing, Boise State University, Boise, ID, USA; ^2^Department of Nursing, Saint Anselm College, Manchester, NH, USA; ^3^College of Nursing, Texas Woman’s University, Denton, TX, USA

##### **Correspondence:** Jane Grassley (janegrassley@boisestate.edu) – School of Nursing, Boise State University, Boise, ID, USA

**Background**

A newborn’s second night is challenging, particularly if it takes place on a hospital mother/baby unit. Mothers are fatigued; babies are unsettled; partners are at their wits’ end; nurses offer the quick fix of the nursery (if the hospital has one) and a bit of formula so mothers can rest; exclusive breastfeeding is sabotaged. Explanations about the second night abound: night nurses are less supportive of breastfeeding; parents have unrealistic expectations about newborn sleeping and feeding patterns; visitors disrupt mothers and newborns’ ability to rest and breastfeed and over stimulate the infant. While any of these might provide an accurate explanation, it is only through listening to the voices of mothers and nurses that we can gain a better understanding of the challenges of the second night.

**Methods**

Feminist scholarship emphasizes the primacy of listening to women’s voices in understanding life experiences. One path is through dialogue, which involves “a *mutual* exchange of knowledge based on one’s experiences in and of the world. Through such collaboration, people are able to transform their lived experiences into knowledge” [1]. This paper presents a dialogue between a mother and a mother/baby nurse working the night shift. The presenters, using research findings from two studies, created the content of the dialogue: an institutional ethnography of the night shift and an analysis of qualitative data from a postpartum depression study. The three presenters played the roles of mother, nurse, and narrator. All involved institutions granted human subjects approval for the studies.

**Findings**

Mothers and nurses shared common and differing perspectives of the challenges of the second night. The challenge of mothers’ fatigue was a shared concern; however, they posed differing perspectives about its source. The nurses focused on the presence of visitors as a cause for fatigue, while mothers saw rooming–in as the source of not sleeping. The nurses spoke of the challenges about the time and effort taken to help a mother and newborn having difficulties breastfeeding and concerns about their other patients. Mothers sensed when nurses seemed “too busy” and were reluctant to ask for help. Mothers also expressed concern that postpartum and infant care education was designed around nurses’ priorities rather than mothers’ needs.

**Implications**

The second night presents challenges to mothers and nurses. Listening to one another’s perspectives about these challenges has the potential for creating supportive hospital environments that promote exclusive breastfeeding while helping families develop confidence in their ability to care for their newborns. Presenting the analysis of two studies with the spoken words of mothers and nurses in a dialogue allowed the audience to hear and see a range of expressed emotions including fear, exhaustion, frustration, concern, empathy and compassion. Dialogue made qualitative data come alive and promoted a deeper understanding of mothers’ needs and expectations following childbirth and nurses’ work in caring for and supporting new mothers. Hospital policies regarding visitors and more focus on assessing and addressing mothers’ fears and concerns could help lessen the challenges of the second night.

**References**

1. Hernan, A. (2015, July 19). 6 strategies every feminist needs for effective change-making dialogue, Everyday Feminism (para 23) Retrieved from http://everydayfeminism.com/2015/07/effective-dialogue-strategies/

## A13. The role of lactation consultants in two council breastfeeding services in Melbourne, Australia – some preliminary impressions

### Jennifer Hocking, Pranee Liamputtong

#### School of Science and Health, Western Sydney University, Richmond, Australia

##### **Correspondence:** Jennifer Hocking (jenhock13@gmail.com) – School of Science and Health, Western Sydney University, Richmond, Australia

**Background**

Australia has 2,165 IBCLCs (International Board Certified Lactation Consultants) – the most per capita of population of any country in the world. More than 90 % of these have had some midwifery training.

Australia also has one of the world’s highest breastfeeding initiation rates – last measured at 96 % in 2010. As in most industrialised nations, breastfeeding rates quickly decline after the baby is one week old, with exclusive breastfeeding rates at “less than 6 months” equal to 15 % [1]. Australian rates of breastfeeding initiation and duration are reflective of maternal education levels and socio-economic status. Lactation Consultants are specifically qualified to work with breastfeeding women and their families and yet we know little about their work, particularly in the Australian context. One Australian study conducted interviews with 12 Lactation Consultants in Melbourne, Australia [2]. Their conclusion that Lactation Consultants were “fluid experts” stressed the negotiation that Lactation Consultants do in their everyday work within a post- modern work environment: they constitute a bridge between the medical needs of the institution for which they often work and the maternal needs of their clients. Jennifer Torres’ more recent ethnographic work in the United States also notes that they “medicalise to de-medicalise” in their work – providing care for women in a humanistic way, within the often biomedical contexts of their workplaces, and against a background of their own comprehensive scientific knowledge [3].

**Approach**

Our ethnographic research involved conducting participant observation of lactation consultants’ work across their sphere of practice in Melbourne, Australia. After periods of observation, we conducted semi-structured interviews with the Lactation Consultants around the question: “how do you view your role as a Lactation Consultant”?

**Findings**

This paper presented work from our observation of Lactation Consultants working in breastfeeding services in two specific local councils – both of which are fast- growing municipalities with young populations, significant ethnic diversity and relatively low socio-economic standing. Both have low breastfeeding initiation and duration rates relative to the state average. We were interested in both the macro and micro influences on provision of support – models of care, professional support of Lactation Consultants and their perception of how these variables impact on their work with these vulnerable groups. These two council services provide an interesting contrast to each other: one can be described as embodying “exciting potential” with two clinicians committed to providing relevant support to their clients and attempting to strengthen their community’s attitudes to breastfeeding. The other service is described as “constrained” where service structures and management approaches limit both the models of care and autonomy of the clinicians.

**Implications**

Lactation Consultants want to be able to practice with some degree of autonomy but in a society that is ambivalent at best about breastfeeding, they also need support to move beyond working “at the bottom of the cliff”. With this support they are more likely to be able to have an impact on their wider community’s breastfeeding culture.

**References**

1. AIHW. (2011). *2010 Australian National Infant Feeding Survey: indicator results*. Canberra: Australian Institute of Health and Welfare

2. Carroll, K., & Reiger, K. (2005). Fluid experts: lactation consultants as postmodern professional specialists. *Health sociology review, 14*(2), 101-110.

3. Torres, J. M. C. (2014). Medicalizing to demedicalize: Lactation consultants and the (de) medicalization of breastfeeding. *Social Science & Medicine, 100*, 159-166. doi:10.1016/j.socscimed.2013.11.013

## A14. Integrating social marketing and community engagement concepts in community breastfeeding programs

### Sheree H. Keitt, Harumi Reis-Reilly

#### National Association of County and City Health Officials, Washington, DC, USA

##### **Correspondence:** Sheree H. Keitt (skeitt@naccho.org) – National Association of County and City Health Officials, Washington, DC, USA

**Introduction**

Although breastfeeding rates in the United States have risen, persistent disparities remain in African American and other underserved populations. Rates among African Americans have consistently fallen short of the national objective in initiation, duration, and exclusivity. According to the United States Department of Health and Human Services [1], there are many barriers to these mothers achieving optimal access to supportive services, including lack of knowledge, social norms, and social support. Additionally, recruitment practices and meeting locations can aide in the lack of participation of mothers. Community-based organizations are key facilitators in community-level interventions, but often lack social marketing skills and community engagement plans to engage mothers into their services. The integration of social marketing and community engagement in health promotion planning is essential for any program to have success in recruitment and retention.

**Discussion**

This presentation offered a perspective on how breastfeeding support programs can effectively apply social marketing concepts and community engagement principles to health promotion planning in order to develop critical partnerships with stakeholders and the recruitment of families in their target population. Practical ways to integrate the 4 P’s of marketing into health promotion planning to address breastfeeding services were described, for example: support groups and drop-in clinics (Product) that consider common challenges of the overall structural barriers (Price) of underserved women; and the importance of enhancing local partnerships (Place) and cultural tailored messaging to the target population (Promotion) to boost program recruitment and attendance. Community engagement principles, informed by public health behavior theory, were discussed as a mechanism for understanding the dynamics of social networks and relationship building in in the recruitment of minority and underserved women into community-level programs. The Social Ecological Model informed the discussion on the effects of environmental and sociological influences on a mother’s perception of and decision to breastfeed. Additionally, the constructs of the Theory of Planned Behavior, which include attitudes, subjective norms, and perceived behavioral control, were discussed as a way of addressing barriers and facilitators related to a mother’s intention to engage in breastfeeding support programs. This session highlighted examples of the implementation of these strategies by grantee organizations from the Centers for Disease Control and Prevention- National Association of County and City Health Officials funded project “Reducing Disparities in Breastfeeding through Peer and Professional Lactation Support”, including local health departments, community-based organizations and local hospitals.

**Conclusion**

Integrating community engagement principles into health program planning is essential for any program to have success in the recruitment and retention of mothers into breastfeeding support programs.

**References**

1. United States Department of Health and Human Services. (2011). *The Surgeon General’s Call to Action to Support Breastfeeding.* Retrieved from www.surgeongeneral.gov/library/calls/breastfeeding/calltoactiontosupportbreastfeeding.pdf

## A15. What happens before and after the maternity stay? Creating a community-wide Ten Steps approach

### Miriam Labbok^^^

#### Carolina Global Breastfeeding Institute, Department of Maternal and Child Health, UNC Gillings School of Global Public Health, University of North Carolina at Chapel Hill, Chapel Hill, NC, USA

^^^Deceased

Dr. Labbok passed away before this supplement was published. We want to acknowledge here the value of her work for decades on behalf of breastfeeding mothers and babies. For further information about this work please contact Catherine Sullivan at CGBI at Catherine_Sullivan@unc.edu.

**Background**

The Baby-friendly Hospital Initiative (BFHI) was launched in 1990/91 by UNICEF/WHO. In 2005/6, stimulated by discussion at a WABA-hosted international meeting in 2002, UNICEF/ WHO decided to update the BFHI guidance. Guidance for expanded BFHI (EBFHI) beyond the maternity setting was developed in recognition that Step 10 was not sufficient for post-maternity support. The “Criteria for Baby-friendly Communities” were developed for the 2010 revised and expanded BFHI materials, clearly noting that this should be comprehensive and not limited to the health system alone. While there have been many efforts to create improved community support, there have not been comprehensive approaches for geo-political units created or piloted for global use.

**Perspective**

Why should a city be interested? Cities wish to attract a motivated workforce that seeks a better chance for lifelong health and development, better school performance, and other achievements provided by breastfeeding. Hence, this provides a public relations opportunity for a city as a healthier, more welcoming community for young families of all races and ethnicities.

**Process**

The process used to develop the pilot approach was started by a small group of interested stakeholders, including the Carolina Global Breastfeeding Institute (CGBI), Chapel Hill Rotary, La Leche League and a faith-based organization. Next, we identified, nurtured, expanded, and convened a larger group of potential stakeholders, in this case: the Mayors’ offices, Chamber of Commerce/Enterprise groups, faith-based groups, the County Health Department, and coordination with the State Breastfeeding Coalition. This larger group then publically launched the effort and continued to identify further potential stakeholders, seeking a fully inclusionary approach. Together we brainstormed the EBFHI components, and developed this draft set of steps necessary for designation.

**Ten Steps for a Breastfeeding-Family-friendly City/Community Ten Steps: Beyond Maternity Care**

1. The city's elected/appointed leadership has a written policy statement, routinely disseminated, supporting breastfeeding.
2. The city as a whole provides a welcoming atmosphere for breastfeeding families
3. Optimal breastfeeding is supported by health leadership
4. All pregnant women are informed about the benefits of breastfeeding, as well as about risks of unnecessary formula use, and where to access support as needed, using media, etc.
5. Health care provision is breastfeeding-friendly
6. Non-health system breastfeeding support groups and services are fully available in the community, including WIC, LLL, IBCLCs, etc.
7. The business, faith-based and social organizations in the community/city as a whole provide a welcoming atmosphere for breastfeeding in public.
8. Local economic development, as well as businesses and not-for-profit organizations, follow the principles of the Code of Marketing
9. The WABA Maternity Care or the US Business Case for breastfeeding is distributed and promoted by the government and the Chamber of Commerce
10. K-12, colleges and universities are encouraged to include breastfeeding-friendly curricula at all levels

The current plan is to launch a website/platform so that all groups working on any one of these steps, or all of these steps, might share their experiences. A temporary site for sharing your actions under each step has been established at: http://www.bffriendlycities.com/.

## A16. #RVABREASTFEEDS: cultivating a breastfeeding-friendly community

### Leslie Lytle (Leslie.Lytle@richmondgov.com)

#### Richmond Healthy Start Initiative, Richmond, VA, USA

**Background**

Richmond, Virginia, is home to a diverse population of approximately 217,853 people within a larger Metropolitan Area of 1,260,029. More than one out of four (26.3 %) of Richmond’s residents live at or below the poverty line. Of the 2837 live births to city residents in 2013, 1664 (58.6 %) were to African-American mothers. In July 2011, a Breastfeeding Commission was convened and charged with developing strategies to increase the number of women in the City of Richmond who breastfeed their children. The Breastfeeding Commission developed four recommendations, one of which was to develop an education/marketing strategy to promote breastfeeding as a cultural norm. Utilizing the Breastfeeding Commission’s recommendations, Richmond Healthy Start Initiative (RHSI) won two consecutive grants through which a coalition, the Richmond Health Action Alliance - Healthy Communities Action Team (RHAA-HCAT) was formed. During World Breastfeeding Week (August 1-7) 2015, the RHAA-HCAT conducted a social media campaign with the goals of 1) promoting breastfeeding as the cultural norm, 2) increasing community support for breastfeeding mothers, and 3) raising awareness about a new law protecting pubic breastfeeding. Campaign costs of $14,100 were covered by different organizations within the RHAA-HCAT.

**Campaign components**

A professional photo shoot of local breastfeeding mothers was conducted, resulting in eleven images representative of Richmond’s diverse community. Several included fathers and family members to represent support for breastfeeding mothers. Images were made into slightly larger-than-life-size cutouts. A graphic designer was hired to create a logo that was colorful and fun, a collateral piece in the form of a fan, and a “Breastfeeding Welcome Here” sticker in English and Spanish. A consultant managed the social media aspect of the campaign, including development of the campaign website, social media strategy, scheduling of posts, and collection of social media data. Community leaders were recruited to serve as “Breastfeeding Champions” during the campaign. Cutouts were strategically placed in 30 locations throughout the campaign, with an emphasis on low-income neighborhoods. Volunteers accompanied the cutouts to share information about breastfeeding, the new public breastfeeding law, and local infant-feeding resources. Citizens were encouraged to take selfies with the cutouts utilizing pre-formatted signs available for download from the website.

**Campaign results**

The campaign received 99,100 engagements on Facebook, Twitter, and Instagram and wide media coverage. Volunteers engaged with approximately 3000 individuals during the campaign. The campaign fan was hugely popular and extremely useful as a conversation starter; 1700 were distributed.

**Next steps**

The #RVA Breastfeeds Campaign focused the energy, talents, and resources of multiple stakeholders on a creative and engaging project. Building on relationships established during the 2015 campaign, RHAA-HCAT members are producing a breastfeeding event through which care providers from multiple agencies will be trained in the use of a research-based breastfeeding education tool. Plans for the 2016 #RVA Breastfeeds Campaign include curating breastfeeding stories gleaned from RVA citizens and the creation of a community-driven mural project.

## A17. Public health vs. free trade: a longitudinal analysis of a global policy to protect breastfeeding

### Mary Ann Merz (merzma@prodigy.net)

#### St. Louis Breastfeeding Coalition, St. Louis, MO, USA

**Background**

The International Code of Marketing of Breastmilk Substitutes (the Code) is a policy to restrict marketing practices in the interest of public health. Adopted in 1981 by 118 countries, the Code was opposed by only one country, the United States, on the basis of free trade. The International Baby Food Action Network (IBFAN) has conducted a survey of the implementation status of the Code by countries every three years since it was adopted. Despite the Code and its own internal policies, the baby food industry aggressively promotes their products. It is important to study the implementation of the Code to understand its usefulness in protecting breastfeeding around the world.

**Method**

This study utilized an ecological research design using multilevel growth curve modeling techniques in which Code status at nine time points (1986-2011) were nested within 197 countries. The research hypotheses were: (1) Code Status increases over time and differs by country, and (2) implementation of the Code is associated with the developmental status of the country, women’s education, and infant mortality. Data analysis was completed using the statistical package R.

**Results**

The average level of Code implementation across all countries was 4.78 (voluntary policy) on a scale of 1 (no action) to 9 (law). Both the level of implementation and the rate of change varied across countries. The global pattern of implementation was a positive, curvilinear trend. The three predictors each contributed to model fit. Middle-income countries had a slightly higher level of implementation than high-income countries. Code status was lowest for the least developed countries over time; however, this group also had the highest percentage of countries with a legally enforceable Code in 2011.

**Conclusion**

The good news is that implementation of the Code is increasing over time across countries. The big question is whether it is possible for public health and free trade to coexist within the current global context of free trade agreements. Although not well known, the preamble of the World Trade Organization states that “free trade should contribute to an improvement in the standard of living,” not merely as a goal in itself. Likewise, national governments, which have the sole authority to enforce the Code, also have an interest in protecting economic prosperity.

The Philippines is an example of the struggle to protect breastfeeding against the power of the multinational baby food industry’s threats of trade sanctions. Justice Leila de Lima ruled in favor of a Department of Health policy prohibiting trademarks with claims that undermine breastfeeding, stating that it was “reasonable regulation of an industry which affects public health.” She further stated that “trademarked claims can be regulated for the greater good”. These findings suggest that a continued effort to implement the Code into law at the country level is a viable strategy to protect breastfeeding from aggressive marketing practices. It also highlights to the value of having women in positions of authority.

## A18. Legislative advocacy and grassroots organizing for improved breastfeeding laws in Virginia

### Kate Noon (katenoonlcsw@gmail.com)

#### VA Alliance for Breastfeeding Laws, Richmond, VA, USA

**Introduction**

Breastfeeding protections are key to increasing breastfeeding success. Laws are needed to ensure breastfeeding rights, raise awareness and contribute to the normalization of breastfeeding. As of 2014, Virginia was one of only three states without a law protecting the right to breastfeed in public (a 2002 law protected breastfeeding only on property owned, leased, or controlled by the Commonwealth). Lack of legal protection leaves mothers, children, and families open to discrimination in which they may be denied access, accommodations, goods, and services.

**Materials and methods**

The goal of the VA Alliance for Breastfeeding Laws, founded in 2014, is to improve breastfeeding laws and protections in Virginia. By 2016, the Alliance had grown to over 780 breastfeeding supporters. Constituents worked with legislators to draft House Bill 1499 and Senate Bill 1427, protecting a mother’s right to breastfeed in any place where the mother is lawfully present. Through education and empowerment of individuals, organizers activated constituents across the Commonwealth and help them engage in the legislative process. Traction was quickly gained and momentum grew in support of the legislation through a combination of direct lobbying, grassroots lobbying, telephone calls, emails, and press coverage. Testimony was collected documenting instances in which women, children, and families experienced discrimination in public places simply for feeding children in the biologically intended manner; this testimony was presented in-person at committee meetings and by writing individual legislators. Business cards and short bullet-pointed handouts about the need for the law was distributed to all 140 Virginia legislators; individuals engaged in direct lobbying in the General Assembly “branded” themselves by wearing lapel pins or stickers with the international breastfeeding symbol. Additional support was developed through collaboration with stakeholders including: American Academy of Pediatrics, American College of Nurse Midwives, American Civil Liberties Union, National Women’s Law Center, March of Dimes, Women Matter, The Catholic Conference, and The Family Foundation. Use of positive media coverage was a successful strategy in cultivating public awareness and public support of the legislation. The grassroots efforts received substantial media coverage, including articles in The Washington Post and Huffington Post.

**Results**

With broad bipartisan and public support, the Virginia General Assembly unanimously passed both bills. Governor Terry McAuliffe incorporated the bill signing ceremony into a reception at the Governor’s Mansion to honor Women’s History Month. Throughout the state, “Nurse-Out” events celebrated the enactment of the new Right to Breastfeed in Public Places law.

**Next steps**

The VA Alliance for Breastfeeding Laws remains active year-round, and annual “Breastfeeding Lobby Day” activities will continue each January at the start of the General Assembly session to keep attention focused on advancing breastfeeding related legislation and policy. Ongoing legislative efforts seek to improve state-level policy elated to workplace pumping protections, eliminate employment discharge on the basis of pregnancy, childbirth, lactation or related medical conditions, create parental leave tax credits for small businesses, and prohibit coercion of pregnant women regarding options related to childbirth. Additionally, Virginia is working to become the first state to have a specialty license plate in support of breastfeeding.

## A19. Breastfeeding and the rights of incarcerated women

### Krista M Olson (kolson@familysupporthawaii.org)

#### Family Support Hawaii, Kealakekua, Hawaii, HI, USA

**Introduction**

Given current evidence regarding the risks of not breastfeeding, health care providers have a responsibility to advocate for alternative sentencing and family reunification to support the lactation goals of incarcerated mothers. When mother-baby separation is unavoidable, lactation professionals can advocate for effective supports to facilitate the provision of breastmilk by inmates who desire to express milk for their infants. Identifying optimal individual breastfeeding outcomes may be complicated when mothers have a history of poly-substance use, when geographic distance makes transport of breastmilk difficult, or when infants are in state foster care. Despite the unique obstacles experienced by mothers in the criminal justice system, those who find adequate support to meet their own breastfeeding goals describe significant physical, social and emotional benefits for themselves and their families.

**Perspective**

Persistent racial and social inequities in rates of breastfeeding in the United States overlap in disconcerting ways with women’s experience in the nation’s criminal justice system. Breastfeeding advocates are familiar with socio-economic and racial disparities in US breastfeeding outcomes. Demographics of the growing number of women incarcerated in the US show a distressingly familiar pattern of disparities. Many of the nation’s most vulnerable infants begin life with the added burden of early breastfeeding cessation when they are separated from mothers who are jailed or imprisoned. Incarcerated mothers are unable to reap physical and mental health benefits associated with lactation because they are denied access to both direct breastfeeding and other means of milk removal. As the population of female inmates in the US increases considerably faster than males, we face urgent questions about which rights are surrendered when incarcerated. Tremendous inconsistencies exist among policies addressing the right to breastfeed or provide breast milk when in jail or prison. Many mothers find there simply are no policies, and accommodations come too late or are too fragmented to protect breastfeeding. Most pregnant women entering the justice system, as well as those who enter as currently breastfeeding mothers, face short and long term health risks of disrupted lactation.

**Recommendations**

Lactation professionals bring critical insight to shaping clinical guidelines for the care of women prisoners, and to broader discussions regarding sentencing and prison reform. Jails and prisons need accurate clinical information to address acute concerns and long-range goals for the thousands of US female inmates who are either pregnant or have given birth within the past year at the time of arrest. Needs may include preventing engorgement and mastitis, protecting or establishing milk supply, addressing hormonal changes associated with abrupt weaning, or safely suppressing lactation in accordance with individual maternal goals. Clinicians can educate colleagues in the criminal justice system about the impact of early breastfeeding cessation and advocate for alternatives that protect lactation, strengthen families, and decrease recidivism. Alternatives that have shown promise include deferred sentencing, addiction treatment programs, community-based sentencing and prison nursery programs that keep children with their mothers.

## A20. Barriers and support for Puerto Rican breastfeeding working mothers

### Ana M. Parrilla-Rodríguez^1^, José J. Gorrín-Peralta^1^, Melissa Pellicier^2^, Zeleida M. Vázquez-Rivera^1^

#### ^1^Maternal and Child Health Program, School of Public Health, University of Puerto Rico, San Juan, Puerto Rico; ^2^Coalición para la Lactancia Materna en Puerto Rico, San Juan, Puerto Rico

##### **Correspondence:** Ana M. Parrilla-Rodríguez (ana.parrilla@upr.edu) – Maternal and Child Health Program, School of Public Health, University of Puerto Rico, San Juan, Puerto Rico

**Introduction**

Women make up 52 % of the general population in Puerto Rico and 45 % of the island's total labor workforce and 54 % of public-sector workforce. 30.8 % of all families in Puerto Rico are led by women [1]. Puerto Rico has had a Working Mothers Act since 1942 that provides an eight-week maternity leave at full pay for employees working in Puerto Rico and, since 2002, a law for breastfeeding mothers granting them paid breastfeeding or pumping breaks during working hours. A woman who returns to work after maternity leave has a right to breastfeed her baby or express milk for one hour each full working day. Unfortunately, employees who work less than 7.5 hours daily are not covered by these provisions. This hour may be divided into two 30- minute breaks, or three 20-minute breaks.

**Methods**

This is the first study designed to identify barriers and supportive factors for breastfeeding working mothers. A survey of nine questions answered by 249 working breastfeeding women was conducted during the 2015 Breastfeeding Week to identify barriers and supportive factors for breastfeeding during working hours.

**Results**

Around two thirds of the mothers (65.1 %) were from 26-35 years of age. 32.7 % of the babies were 0-6 months of age, and 27.2 % were 7-12 months of age. 88 % of the participants were breastfeeding. Among the 26 mothers who were not breastfeeding at the time of the survey, 42.3 % had breastfed for more than 24 months. Identified barriers to breastfeeding included: maternity leave less than six weeks (18.4 %); inadequate support from husband or partner (12.4 %); unavailability of pumping station (35.9 %); overfeeding of baby in day care (17.5 %); 15 minutes or less twice a day granted for pumping (19.7 %); and no substitute available during pumping breaks (27.8 %). Assorted barriers, for a total of 37.2 %, included: inadequate pumping station facilities; lack of knowledge of the law to protect the breastfeeding working mother by the employer; high levels of stress at work, and scanty milk output at extraction. The majority of working mothers reported that their most important support for breastfeeding came from relatives and co-workers: husband or partner (32.1 %), co-workers or employer (20.9 %), family (11.2 %), pediatrician (6.7 %) and friends and day care personnel (14.2 %).

**Conclusions**

There are presently over 25 laws in Puerto Rico related to breastfeeding. In spite of these legal tools women face many barriers which make breastfeeding and working difficult. Present laws seem to be insufficient for fulfilling optimal breastfeeding goals. It is necessary to review the legal framework and create mechanisms to monitor compliance. Employers must be educated on the existence of these laws and to the need to comply with them.

**References**

1. Kantrow, M. 2014. Women head Puerto Rico’s public sector workforce. Retrieved from:http://newsismybusiness.com/puerto-sector-workforce/

## A21. Pumping at work: a daily struggle for Puerto Rican breastfeeding mothers in spite of the law

### Melissa Pellicier (Mpellicier@gmail.com)

#### Coalición para la Lactancia Materna en Puerto Rico and Fundación Puertorriqueña para la Protección de la Maternidad y la Niñez, Promani, San Juan, Puerto Rico

**Background**

In Puerto Rico women are 45 % of the total labor workforce [1]. Since 2000 Puerto Rican breastfeeding working mothers are entitled to paid breaks for breastfeeding or pumping at their work place. However, fifteen years have passed and mothers are still facing serious difficulties when employers use all sorts of mechanisms for not complying with the law and continue to discriminate. State and local federal courts are recently deciding on these issues. Cases reveal ongoing violations of the law, discrimination against women and violations of basic human rights.

**Approach**

Three state court cases from the Court of Appeals were studied and analyzed to understand the context, facts and grounds of the court’s decisions. Plaintiffs described all sorts of problems including inadequate pumping station facilities, lack of knowledge of the law, harassment, discrimination, retaliation, reduced milk output at extraction and emotional and/or physical distress.

**Findings**

The entire family circles suffered the consequences of these events. In two of three cases plaintiffs prevailed. We found that foundations and discussions turned around the rights of intimacy and privacy, but mostly around the women’s right to decide to breastfeed or not. Likewise, state laws and policies in support of breastfeeding were key elements for judges to understand and articulate the issues at stake. On the third case, the court disregarded the discussion of the human rights mentioned and above all, the laws and policies designed and enforced since 1995 to support, protect and promote breastfeeding in Puerto Rico. It resulted in a narrow analysis of facts and the law. Moreover, the court ignored the variety of tactics that an employer is able to impose to discriminate against women who decide to breastfeed. Finally, the court overlooked to discuss the short and long term consequences of denying the right to breastfeed for the mother and the baby. Recently the Supreme Court of PR overruled the Court of Appeals decision.

**Conclusion**

In a country where maternity leave lasts, at the most, only twelve weeks, laws to facilitate breastfeeding and pumping are crucial for women who decide to keep breastfeeding. However, laws remain insufficient in light of many other strategies to discriminate at the work place. It is necessary to revise these laws to incorporate recent jurisprudence and experience. Information gathered from women, agencies, non-governmental organizations, employers and advocates shall be used to educate co-workers to prevent discrimination. Mechanisms to monitor compliance are needed to allocate responsibilities in time, so the women are not forced to decide between breastfeeding and work.

**References**

1. Working Women in Puerto Rico Earn Slightly More Than Men. (2014). Puerto Rico Report. Retrieved August 9, 2016 from: http://www.puertoricoreport.com/working-women-in-puerto-rico-earn-slightly-more-than-men/#.V6dzRY6zNEA

## A22. “I saw a wrong and I wanted to stand up for what I thought was right:” a narrative study on becoming a breastfeeding activist

### Jennifer L. Pemberton (jpemberton@ccbcmd.edu)

#### Behavioral Sciences, Community College of Baltimore County, Maryland, USA

**Introduction**

The purpose of this narrative study was two-fold: (a) to examine how breastfeeding mothers learn they are members of a marginalized group; and (b) to investigate how some of these mothers move from marginalization to activism. This study was grounded in two interconnected theoretical frameworks: critical feminism (also with attention to embodied learning) and women’s emancipatory learning in relation to breastfeeding and activism.

**Participants and methodology**

The 11 participants in the study were purposefully chosen according to criteria related to the study’s purpose; their diversity in age, race/ethnicity, sexual orientation, religion, and educational background. Data collection included narrative semi-structured interviews, which were co-constructed between the researcher and the participants, and researcher-generated artifacts. Both narrative and constant-comparative analysis were used to analyze the data.

**Findings**

There were three sets of findings that emerged from the data. First, those related to the marginalization of breastfeeding mothers indicate that their marginalization is manifested in: negative views of breastfeeding in public; lack of breastfeeding support of some health professionals; the commercial formula industry; and returning to employment. Second, the findings regarding how they learned to be activists indicate they did so: by becoming conscious of marginalization; through mentoring, networking, and collaboration; through sometimes leveraging men’s power and support; and through social media and technology. Third, the findings regarding what they learned from being activists center on: seeing activism as a continuum; perspective-taking; learning leadership skills; and claiming their own empowerment.

**Implications and future research**

The findings from this study have implications for both theory and practice. Related to adult learning theory, this study offers new insight into the role of embodied learning as part of women’s activist learning. The public health field can glean from this study how to better educate women not only on breastfeeding, but also on public health issues in general by fostering collaboration and connection, and adopting insights from feminist pedagogy. Further, integrating activism and emancipatory learning into the curriculum for health care professionals can help them not only in their efforts at patient education, but in their own activist efforts for healthcare. This study demonstrates the need for further research. A limitation of this study is the homogeneity of participants in regard to socioeconomic status; more specifically, the participants represented middle and upper middle class backgrounds. Future research should consider the implications of socioeconomic status on activism with a focus on how to create conditions that encourage women with socioeconomic challenges to become involved in activism. Furthermore, future research ought to investigate how feminist pedagogy and principles of women’s learning can create the conditions necessary for some of the most marginalized groups – such as people living in developing countries or those with disabilities – to recognize their own agency so they can be empowered to work for social change.

## A23. Peer breastfeeding support: advocacy and action

### Catherine McEvilly Pestl (catherinepestl@gmail.com)

#### Lactation Consultant, Private Practice, Toronto, Canada

**Introduction**

Lack of social support is a key factor associated with poor breastfeeding outcomes. Peers strongly influence a woman’s intention to breastfeed. Advocacy for the creation of breastfeeding support groups in diverse communities has increased awareness of the importance of peer support. Community engagement offers the potential for leadership; roles arise for women to make a meaningful impact within their community. Communities come in many different forms and locations, including physical communities, spiritual or faith-based communities, cultural communities and priority (or at-risk) communities. These community-based breastfeeding support groups offer peer support in populations with lower rates of breastfeeding in Toronto, Canada.

**Perspective**

The goal of these groups is to engage populations with lower breastfeeding rates and to advocate for peer breastfeeding support. Being a professional involved with a community can be very different than being a member of the community. As Lactation Consultants, breastfeeding support groups allow us to explore a “peer-to-professional” model, which allows for optimum exposure to breastfeeding support. This community-based research explores the creation of peer support groups in community settings, as well as the importance of working in unison to provide support in a format that reaches mothers and empowers from within. Issues of access and usage are explored, as are the creation and sustainability of these model groups.

**Impact**

Pre- and post-evaluations were completed to assess change during all-staff training as well as during the drop-in support group sessions. The introduction of breastfeeding as well as the importance of its support and protection to all community partners in the form of staff training resulted in an increase of confidence and knowledge of breastfeeding support. The women attending these groups reported an increase in confidence and knowledge of normal newborn breastfeeding behaviour. These women also reported that they intended breastfeeding longer than planned as a result of attending these support groups.

## A24. Good intentions: a study of breastfeeding intentions and postpartum realities among first-time Central Brooklyn mothers

### Jennifer Pierre, Philip Noyes, Khushbu Srivastava, Sharon Marshall-Taylor

#### Brooklyn District Public Health Office, Center for Health Equity, New York City Department of Health and Mental Hygiene, Brooklyn, NY, USA

##### **Correspondence:** Jennifer Pierre (jpierre4@health.nyc.gov) – Brooklyn District Public Health Office, Center for Health Equity, New York City Department of Health and Mental Hygiene, Brooklyn, NY, USA

**Background**

The health benefits of breastfeeding for both mother and infant are well-documented. The American Academy of Pediatrics recommends exclusive breastfeeding for at least six months. In 2011, while most mothers in New York City (NYC) initiated breastfeeding (89 %), only 32 % exclusively breastfed in the first few days of life, and 14 % at 6 months. Even fewer low income women and women of color exclusively breastfeed [1]. Only 5 % of mothers participating in the Women, Infants and Children (WIC) Program, were exclusively breastfeeding their babies at six months. In the high poverty neighborhoods of Bedford-Stuyvesant and Brownsville this was even lower at 4 % and 1 % respectively [1]. The Breastfeeding Empowerment Zone (BFEZ) project is a W. K. Kellogg Foundation grant-funded initiative of the NYC Department of Health and Mental Hygiene (DOHMH) to address racial and health inequities in breastfeeding, and improve infant health by increasing breastfeeding support. This qualitative study, conducted between February and September 2014, was developed to deepen the understanding of Black and Hispanic women’s experiences with infant feeding decision-making to inform the BFEZ project.

**Methods**

First time pregnant Black or Hispanic women who lived in Bedford-Stuyvesant or Brownsville for at least 2-years were recruited for this qualitative study. Women were interviewed twice. The initial interview (18-37 years; n = 19) took place during their third trimester and women were asked about their infant feeding plans. During their second interview, at least 4 weeks postpartum, women (n = 17; 2 lost) shared their infant feeding experiences. Key informants (n = 10), fathers and grandmothers, were also interviewed to provide perspectives on infant feeding influences. Researchers reviewed, coded and analyzed interview transcripts for major categories and themes.

**Results**

The majority of women planned to breastfeed. Many women had been formula-fed as babies and had mostly been exposed to formula-fed babies. However, they felt strongly that breastfeeding was the healthier option for their babies due to information learned from health care providers, research and media sources. Unanticipated circumstances altered breastfeeding plans for many. Emergency C-Sections and postpartum illnesses of mother and/or baby resulted in many babies being fed formula in the hospital. Latching problems, perceived low breast milk production, and pain from faulty latch or engorged breasts became deterrents to breastfeeding. The added complication of returning to work or school with no facilities for pumping and storing breast milk, and the discomfort felt by many about public breastfeeding, soon caused many mothers to introduce formula. A few women were able to overcome their challenges and successfully breastfeed. These women were buttressed by support from several quarters—their partners, mothers, hospital staff and other health care providers—who provided continuous support and practical, hands-on interventions.

**Conclusions**

Unexpected challenges in childbirth can derail plans for breastfeeding. For mothers in high poverty neighborhoods, breastfeeding education, practical hands-on help from hospital staff, and support from other health care providers, partners, family members and employers is often needed to help achieve their goals for exclusive breastfeeding.

**References**

1. Srivastava K, Mulready-Ward C, Noyes P, Felida N. Breastfeeding Disparities in New York City. Epi Data Brief, New York City Department of Health and Mental Hygiene. August 2015; 57. Available from: https://www1.nyc.gov/assets/doh/downloads/pdf/epi/databrief57.pdf. Accessed April 10, 2016.

## A25. Women describing the infant feeding choice: the impact of the WIC breastfeeding classes on infant feeding practices in Ionia, Michigan

### Jennifer Proto^1,2^ (jproto@elon.edu), Sarah Hyland^2^ Laurie Brinks^2^

#### ^1^Public Health Associate, Office for State, Tribal, Local and Territorial Support, Centers for Disease Control and Prevention, Atlanta, GA, USA; ^2^Ionia County Health Department, Ionia, MI, USA

**Significance to public health**

Although breastfeeding initiation rates among mothers in the Program for Women, Infants, and Children (WIC) in Ionia, Michigan (78 %) are about 20 percent higher than WIC national averages (63 %) [1]. Ionia WIC participants are at risk for low breastfeeding rates and poor health outcomes for their children and themselves^.^

**Background and rationale for the project**

Data from surveillance of breastfeeding rates in Ionia County lacks sufficient information about how women make their infant feeding decisions. This project evaluated: (1) the impact of WIC breastfeeding classes on women’s infant feeding decisions in the experimental and control groups; and (2), the “Bring Your Baby to Work” program in which a breastfeeding peer counselor brought her baby to the breastfeeding classes in the experimental group. It was hypothesized that having a baby in the class would make women more likely to breastfeed.

**Methods**

Sixty-two sets of pre- and post-surveys were administered to mothers enrolled in WIC’s breastfeeding classes with (experimental group n = 31) and without (control group n = 31) the breastfeeding peer counselor’s baby. The surveys evaluated family members’ influence, the child’s father’s support, and, in the experimental group, the presence of a baby in the class on breastfeeding decisions. Spearman’s Rho and Gamma tests were run to assess correlations.

**Results**

In both groups the classes helped women to make infant feeding choices. Having the breastfeeding peer counselor’s baby in the class did affect infant feeding decisions in the experimental group, but breastfeeding did not change in the control group. In fact, women in the control group were more influenced by the class. For the experimental group the class’s impact on the decision to breastfeed and women feeling able to make an infant feeding choice were moderately correlated. There was also a strong correlation between women finding the baby’s presence helpful in learning about breastfeeding and then deciding to breastfeed. Women who found the class influential in their breastfeeding decision also found the baby’s presence helpful in learning about and deciding to breastfeed. The decision to breastfeed was moderately correlated with family members’ influence and the child’s father’s support. For the control group the class’s impact on the decision to breastfeed and women feeling able to make an infant feeding choice was strongly correlated. There was also a moderate correlation between women planning on breastfeeding and women finding the class influential in their decision and a strong correlation between women planning on breastfeeding and feeling empowered by the class to do so. Finally, there was a weak correlation between the decision to breastfeed and the family’s influence on the breastfeeding decision.

**Implications for practice**

Findings suggest that while the WIC breastfeeding classes, including having a baby present in the class, may play some role in women’s infant feeding choices, women value the child’s father’s and family members’ support and influence. To increase breastfeeding rates, Ionia WIC should look to involve these members in their breastfeeding classes.

**References**

1. National WIC Association. *How WIC impacts the people of North Carolina*. Available at: https://s3.amazonaws.com/aws.upl/nwica.org/northcarolina2016.pdf. Accessed February 12, 2014.

## A26. Local and state programs and national partnership to reduce disparities through community breastfeeding support

### Harumi Reis-Reilly^1^, Martelle Esposito^2^, Megan Phillippi^3^

#### ^1^National Association of County and City Health Officials, Washington, DC, USA; ^2^National WIC Association, Washington, DC, USA; ^3^Association of Maternal & Child Health Programs, Washington DC, USA

##### **Correspondence:** Harumi Reis-Reilly (hreis-reilly@naccho.org) – National Association of County and City Health Officials, Washington, DC, USA

**Introduction**

Breastfeeding is one of the most effective measures mothers can take to prevent disease and protect the health of infants. According to the CDC, 79 % of newborn infants initiate breastfeeding with 49 % and 27 % breastfeeding for six and twelve months, respectively [1]. Only 19 % of infants are exclusively breastfed for the first 6 months of their lives. African American women have the lowest breastfeeding initiation and duration rates of all racial and ethnic groups. In 2011, the amount of African American infants ever breastfed was 61.6 %, compared to 81.1 % for whites. The rate of infants being breastfed at 6 months and 12 months was also lower among African American women, 35 % and 16.4 %, respectively, compared to 52.3 % and 28.4 %, respectively, for whites [1]. Research also shows that socio-demographic factors, such as maternal education, are inversely related to the likelihood to begin and continue breastfeeding. These low breastfeeding rates in any population is a public health concern, but it is particularly problematic for African Americans and underserved families because the benefits of breastfeeding for infants and mothers may be more significant in a community that often scores the poorest on nearly every index of public health research using racial comparisons.

**Approach**

The Breastfeeding Public Health Partners (BPHP) group is funded to convene and coordinate among national efforts to increase breastfeeding rates in all communities, specifically targeting African American and underserved populations. Primary partners include the Association of Maternal & Child Health Programs (AMCHP), the Association of State and Territorial Health Officials (ASHTHO), Carolina Global Breastfeeding Institute (CGBI), National Association of County and City Health Officials (NACCHO), National WIC Association (NWA), and United States Breastfeeding Coalition (USBC). Coordination of national activities are focused on three primary strategies, which align with the Centers for Diseases Control and Prevention including maternity care practices (CGBI, ASTHO, and NWA), peer and professional support (ASTHO, NACCHO, and USBC), and workplace policy and accommodation (AMCHOP, ASTHO, and USBC). Through the focus areas, the partners identified four priority activities to address during the 2015-16 year. These priorities included joint advocacy from partner leaders (led by USBC), supporting evidence-practice and building evidence through program evaluation (led by NACCHO and NWA), supporting communities and states roles in providing breastfeeding support, and sharing resources for community, state, and national level breastfeeding support.

**Impact**

The BPHP group increases awareness of evidence-based and innovative activities occurring at the national, state, and local levels to improve breastfeeding rates and ensure inter-agency communication and collaboration to promote breastfeeding support efforts. Leveraging strengths of each national organization, by peer learning, sharing opportunities, dissemination of best practices and helping sustaining work in states and communities allows for maximization of federal funds. This partnership not only strengthened the efforts of individual programs, but collectively have an exponentially greater impact on public health outcomes.

**References**

1. Centers for Disease Control, National Immunization Survey, Department of Health and Human Services (2015). Rates of Any and Exclusive Breastfeeding by Socio-demographics among Children Born in 2012. Retrieved on February 27, 2016 from http://www.cdc.gov/breastfeeding/data/nis_data/rates-any-exclusive-bf-socio-dem-2012.htm

## A27. Beyond black breastfeeding week: instagram image content analysis for #blackwomendobreastfeed/#bwdbf

### Cynthia L. Sears^1^, Delores James^1^, Cedric Harville^1^, Kristina Carswell^2^

#### ^1^University of Florida, Gainesville, FL, USA; ^2^UF Health-Shands Hospital, Gainesville, FL, USA

##### **Correspondence:** Cynthia L. Sears (csears@ufl.edu) – University of Florida, Gainesville, FL, USA

**Background**

Recent nationally representative survey data shows that Instagram is the preferred social media platform among African Americans, with 38 % of African Americans preferring Instagram, compared to 34 % of Hispanics and 21 % of whites [1]. “Black Women do Breastfeed” originated as a blog written by founder Nicole Sandiford to highlight stories and images of Black mothers who had breastfed or were currently breastfeeding their children. The focus has shifted to the Black Women do Breastfeed Facebook page, which at the time of this writing has over 96,000 followers.

**Methods**

In this study, we used existing research and theory on the use of image-based social media, specifically Instagram, to describe the conceptualization of African American breastfeeding. This research study employed a combination of thematic and content analysis to explore the relationship between the users posting content related to African American breastfeeding and the images that comprise the posted content. The search of Instagram for publicly accessible images associated with the relevant hashtags (#BWDBF, #BlackWomendoBreastfeed) was conducted on June 2, 2015 using the web application hashatit.com. Images were coded to examine visual content (image/video, advertisement, infographic), text-to-image ratio, users, date/time, caption, number of likes, @ mentions, and interaction via comments. We used a grounded approach to thematize and categorize images to identify content categories.

**Results**

A total of 615 images were obtained from the initial hashtag search. From the initial sample, 79 of the urls were no longer active because the image had either been hidden from public view or deleted by the user, leaving a sample of 534. A duplicate search eliminated 26 duplicate urls, leaving a final sample of 508 images. 215 unique users employed one or both of the hashtags. The Instagram users who used these hashtags were predominantly black (91 %, n = 195) and under the age of 30 yrs. (n = 314, 62 %). The majority of images (n = 456, 90 %) were posted by single users; only 30 images were posted either by government or nonprofit agencies (6 %).

**Conclusion**

There was a notable lack of presence by both nonprofit and government organizations, suggesting that there is a gap in the low-/no-cost, easily accessible social marketing channels that will be filled by commercial marketing if this platform is not appropriately harnessed by breastfeeding advocates and educators. Future research directions should examine comparisons between artificial baby milk marketing strategies on social media platforms and user generated breastfeeding promotion content.

**References**

1. Krogstad, J.M. (2015). Social media preferences vary by race and ethnicity. Retrieved from http://www.pewresearch.org/fact-tank/2015/02/03/social-media-preferences-vary-by-race-and-ethnicity/

## A28. Stakeholder views of breastfeeding education in the K-12 environment: a review of the literature

### Nicola Singletary, L. Suzanne Goodell, April Fogleman

#### Department of Food, Bioprocessing and Nutrition Sciences, North Carolina State University, Raleigh, NC, USA

##### **Correspondence:** Nicola Singletary (Nicola.singletary@gmail.com) – Department of Food, Bioprocessing and Nutrition Sciences, North Carolina State University, Raleigh, NC, USA

**Background**

As part of efforts to increase breastfeeding initiation and duration, educational interventions aimed to increase awareness and positive attitudes towards breastfeeding beginning during the school years are recommended by the World Health Organization and UNICEF UK. Breastfeeding education in the school setting offers the opportunity to introduce the topic to a wide range of students from a variety of socioeconomic and cultural backgrounds enabling them to make informed choices about infant feeding when they become parents. The purpose of this work is to present a review of the literature regarding stakeholder views of breastfeeding education programs in schools and to make recommendations for future work in the area.

**Method**

Articles for review were located by searching online databases and journals using the following keywords in various combinations: (1) breastfeeding, lactation, infant feeding, and (2) youth, children, school, adolescents, teenagers.

**Findings**

This review indicates that the views and knowledge of stakeholders such as educators, students, parents, health professionals, and the public regarding breastfeeding education in the school environment have been the subject of minimal study. Existing research indicates that breastfeeding is being discussed in some school environments but the extent of lessons and the specific messages that teachers communicate and children receive have not been explored. In many cases students appear to be interested in receiving more information about breastfeeding especially if it comes from health professionals or breastfeeding mothers. The majority of teachers are supportive of incorporating breastfeeding education in family and consumer sciences, sexual education, and health classrooms; however, time constraints and limited knowledge of infant feeding recommendations may be barriers to implementation.

**Conclusions**

Research studies about the infant feeding knowledge, attitudes, and experiences of school-aged children over the last three decades have contributed to our understanding of students’ awareness of these topics and of the potential impact of breastfeeding education in the school environment. Existing research offers us information that can be used to develop targeted educational programs at the K-12 levels. These programs should work towards improving awareness of breastfeeding in general, and address areas shown as lacking in public knowledge such as the specific benefits of breastfeeding for the mother, the infant and their relationship. Well-crafted lessons have the potential to increase understanding of the importance of breastfeeding and dispel breastfeeding myths commonly held in society. However, more work is needed regarding the views and knowledge of stakeholders such as parents, educators, health professionals, and the public regarding breastfeeding education in the school environment. Further research in these areas would add to our understanding of how best to implement lessons and curricula in the future.

## A29. “The Breastfeeding Transition”: a framework for explaining changes in global breastfeeding rates as related to large-scale forces shaping the status of women

### Paige Hall Smith (phsmith@uncg.edu)

#### Public Health Education and Center for Women’s Health and Wellness, University of North Carolina at Greensboro, Greensboro, NC, USA

**Background**

In many countries around the world women with higher status, as indicated by social power, education and income, are more likely to breastfeed while women with lower status are more likely to use formula earlier and more often. However the relationship between the status of women and women’s infant feeding decisions changes over time and place. It is important that we deepen our understanding of the relationships between women’s status and breastfeeding so we can ensure that social efforts to improve the status of women and public health efforts to protect, promote, and support breastfeeding are synergistic rather than oppositional. This paper introduces the “The Breastfeeding Transition” as a way of describing changes in global breastfeeding rates related to large-scale demographic changes shaping the status of women.

**Methods**

This conceptualization of The Breastfeeding Transition is based primarily on results from large-scale demographic studies that have assessed infant feeding patterns as well as: residence (urban or rural), educational attainment, income, and employment. The conceptualization of women’s status embedded in The Breastfeeding Transition centers on the idea that with increasing education, income, reproductive control, and urban residence women have more power and control and hence, have higher status and absolutely and relative to men.

**Findings**

The literature suggests three identifiable phases to The Breastfeeding Transition, and I am proposing a 4^th^ that may occur in the future:

1. Phase 1: Lower status of women is associated with higher rates of breastfeeding: this is the historical norm in most societies – most women live in societies where their economic and social status is lower than men’s and most women breastfeed.
2. Phase 2: Higher status of women is associated with lower rates of breastfeeding: alongside urbanization, women’s educational rates increase and more women become employed and breastfeeding declines among women with higher status, while remaining constant for lower status women.
3. Phase 3: Higher status of women is associated with higher breastfeeding: women’s status continues to rise and women of higher status return to breastfeeding while breastfeeding declines among women of lower status.
4. Phase 4: In this projected phase, breastfeeding rates increase for both lower and higher status women.

**Discussion**

This transition is important because it seems to be predictable, global, and results in breastfeeding disparities by race, income, educational attainment and other factors that shape women’s status within/between countries. By increasing our understanding of the factors that shape this transition we are able to promote phase 4, whereby rates of breastfeeding increase across all populations of women while still seeing an increase in the women’s status. Many different social, political, policy and programmatic forces underlie the transitions across the 4 phases, including: the degree of (in)compatibility between women’s productive and reproductive roles; (lack of) regulation of marketing of human milk substitutes; focus and scope of breastfeeding protection, promotion and support activities; costs and benefits of motherhood, caregiving and breastfeeding; social norms and preferences for infant feeding; availability and use of devices of milk expression; and population disparities in gender/social status.

## A30. Breastfeeding, contraception, and ethics, oh my! Advocacy and informed decision-making in the post-partum period

### Alison M. Stuebe^1,2^, Amy G. Bryant^3^, Anne Drapkin Lyerly^4^

#### ^1^Division of Maternal-Fetal Medicine, University of North Carolina School of Medicine, Chapel Hill, NC, USA; ^2^Department of Maternal and Child Health, Gillings School of Global Public Health, University of North Carolina at Chapel Hill, Chapel Hill, NC, USA; ^3^Division of Family Planning, Dept of Ob/Gyn, UNC School of Medicine, University of North Carolina at Chapel Hill, Chapel Hill, NC, USA; ^4^Department of Social Medicine, UNC School of Medicine, and the Center for Bioethics, University of North Carolina at Chapel Hill, Chapel Hill, NC, USA

##### **Correspondence:** Alison M. Stuebe (alison_stuebe@med.unc.edu) – Division of Maternal-Fetal Medicine, University of North Carolina School of Medicine, Chapel Hill, NC, USA

**Background case presentation**

The patient is a 15 year old with a previous miscarriage who gave birth to an extremely premature infant. Committed to breastfeeding, the patient was working with a lactation consultant (LC). On the day of discharge, the LC entered her room just as a medical student and resident were preparing to insert a contraceptive implant. The LC took the medical student aside and asked whether they had discussed possible effects of the implant on milk supply. The resident joined the conversation, and the patient asked what they were talking about. The resident advised the patient of “a possibility” that the implant could impact her milk supply, but most likely would not; she also informed her that her chances of becoming pregnant without contraception were high, and a short interval pregnancy would increase her risk of another preterm infant. The patient declined the implant, citing her commitment to breastfeeding. The attending obstetrician expressed concern to us about the LC counseling patients and invoking “personal experience” in the absence of published data as evidence for progesterone-related reduction in milk supply. She expressed concerns that sharing information about a theoretical risk was inappropriate, particularly given the high risk of short interval pregnancy.

**Approach**

This case raises a number of important issues. How should providers discuss the risks of contraception during breastfeeding, particularly in the absence of definitive evidence of the effect of hormones on lactation? What are providers obligated to disclose, and what interests and implications – if any – should we consider when sharing inconclusive data? Does counseling on contraceptives and breastfeeding vary with the age/race/income/education of the patient – and should it? What are challenges to informed decision making in the postpartum period and how should we address them? And most importantly, how do we help women make the right decision for themselves, based on their own priorities and preferences? In our panel session, we reviewed what is known about the effects of hormonal contraceptives on lactation, and of lactation on fertility; examined what considerations frame lactation consulting and family planning counseling; explored possible chafe-points at the intersection of advocacy and clinical care; and considered the ethical complexities regarding informed decision-making in the postpartum context. We discussed how promoting health and well-being for the individual woman includes providing support and accurate information to enable her to make informed decisions about feeding her infant and preventing unintended pregnancy.

**Methods**

We worked with participants from the Breastfeeding and Feminism International Conference to develop a shared decision-making tool for contraceptive implant placement in the postpartum period. Participants used a template to develop questions and information to consider when deciding whether to use a contraceptive implant while breastfeeding.

**Next steps**

We will work with participants and other stakeholders to further develop and refine a patient-centered shared decision-making tool for postpartum contraceptive implants and other contraceptives.

**Consent**

The authors have written informed consent from the individual in this case presentation.

## A31. A hard day’s night: juggling nighttime breastfeeding, sleep, and work

### Cecilia Tomori (ctomori1@jhu.edu)

#### Johns Hopkins Bloomberg School of Public Health, Baltimore, MD, USA

**Introduction**

Nighttime feedings play an important role in maintaining mothers’ milk supply and the breastfeeding relationship, especially when feedings are limited during the day. Meeting the infant’s nighttime breastfeeding needs after returning to work, however, can be challenging and may lead to excessive fatigue, poor maternal mental health and premature weaning. My ethnographic research provides unique insight into these important, but often overlooked, challenges and presents an intimate portrait of how families negotiate them.

**Methods**

Results are drawn from a larger ethnographic study of nighttime breastfeeding undertaken with IRB approval between 2006-2008, with follow-up in 2009. The study [1] focused on 18 first-time middle class mothers and their families contextualized by anthropological, historical, and feminist research and analysis.

**Results**

Most new parents found that medical recommendations that babies sleep on a separate sleeping surface made nighttime breastfeeding and sleep difficult. To facilitate nighttime breastfeeding and maximize sleep, most parents brought their infants into bed for at least part of the night. Some parents found regular use of this integrated “breastsleeping” strategy particularly successful, but problematic because of fears of potential dangers and cultural discomfort related to bed sharing. Most others strove to maintain somewhat separate but proximate sleep arrangements, which alleviated these concerns, and reduced fatigue to a degree. Returning to work placed considerable pressures on the breastfeeding relationship because of limited opportunities for breastfeeding and breastmilk expression during the day, and increased fatigue. Participants drew on numerous educational, financial, and relational resources to delay returning to work, reduce work hours, and limit the impact of work on breastfeeding. Proximate sleep arrangements greatly facilitated mothers’ ability to continue breastfeeding despite these challenges. Over time, however, parents encountered increasing pressure from physicians, relatives, and others to reduce or eliminate nighttime feedings, and move babies out of parents’ beds and bedrooms to get them to “sleep through the night” on their own. While most working mothers resisted these pressures, one physician’s advice to remove the baby from the parents’ room for the night ultimately led to premature cessation of breastfeeding.

**Conclusion**

Returning to work shortly after birth presents multiple barriers to the continuation of breastfeeding. Cultural values of independence, reflected in advice to restrict or eliminate nighttime breastfeeding and proximate sleep arrangements, may play a significant, but understudied, role in undermining breastfeeding even among relatively privileged families. In addition to paid family leave, initiatives to revise guidance for infant sleep and nighttime feeding are needed to facilitate the long-term maintenance of breastfeeding and adequate rest for all mothers.

**References**

1. Tomori, C. Nighttime Breastfeeding: An American Cultural Dilemma. 2014. New York, London: Berghahn Books.

## A32. Empowering change in Indian country through breastfeeding education

### Amanda L. Watkins^1^, Joan E. Dodgson^2^

#### ^1^Global Lactation Education Associates, Raleigh, NC, USA; ^2^Maternal Child Health Nursing Research, St. Louis University, St. Louis MO, USA

##### **Correspondence:** Amanda L. Watkins (awnutrition@gmail.com) – Global Lactation Education Associates, Raleigh, NC, USA

**Background**

Native Americans have significant health disparities, including high rates of diabetes, asthma and other chronic illnesses that are decreased when infants have been breastfed for at least 6 months. Although breastfeeding rates vary greatly in Indian Country, the need for evidence-based breastfeeding support and promotion is critical for successful continued breastfeeding. Indian Health Service clinics and tribal services are usually the first line of breastfeeding support for breastfeeding Native families. Our study aim was to determine the effect of breastfeeding education on healthcare providers’ perceived behavioral control related to evidence-based lactation services to mothers.

**Methods**

A pre/posttest survey design with convenience sample of professional and paraprofessional healthcare providers (n = 87) employed by the Indian Health Service or tribal governments was conducted. The target population attended a 45-hour on-line breastfeeding education program offered 6 times between 2013 and 2015. The breastfeeding course focused on educating and supporting breastfeeding families. Perceived behavioral control is a construct within the health motivation Theory of Planned Behavior, which measures a person’s beliefs about their ability to carry out an activity within their environment. The Workplace Perceived Behavioral Control Scale [1] has a self-efficacy (perceived ability to perform evidence-based breastfeeding support) and controllability (perceived ability to implement evidence-based breastfeeding support within work environment) subscales that measure each of these concepts. This construct has 2 components; self-efficacy and controllability. The survey was administered using REDCap web-based survey tool.

**Results**

Participants were predominately minority women (59.9 % American Indian and 29.2 % Hispanic) with 44.6 % having an associate degree or less education. Both professional (n = 49; 56.3 %) and paraprofessionals (n = 38; 43.7 %) responded. Significant improvement in the total Workplace Perceived Behavioral Control Scale and the self-efficacy subscale scores occurred after completion of the course. Participants’ scores on the controllability subscale were not significantly different after the educational program.

**Conclusions**

The unchanged controllability subscale items may suggest areas where the work environment needs change and/or further assessment is required. Of all the possible outcomes of education, behavioral change toward greater evidence-based practice is fundamental, yet, seldom measured in breastfeeding research. These findings raise questions about the infrastructure at participants’ work environments and participants’ willingness to advocate for change in their workplace infrastructure, which was not addressed by this survey. Based on these findings, we propose measuring perceived behavioral control as an educational outcome when seeking changes in practice; it is an effective way to validate the empowering effects of education through changes in self-efficacy and to identify work environment issues hampering use of best practices.

**References**

1. Dodgson, J.E. & Watkins, A. L. Revised The Workplace Perceived Behavioral Control Scale. *An Exploration of Changes in Healthcare Providers’ Learning Outcomes Related to Breastfeeding Support and Promotion Name* [dissertation]. 2015. ProQuest Dissertation Publishing

## A33. Servants and “Little Mothers” take charge: work, class, and breastfeeding rates in the early 20^th^-century U.S.

### Jacqueline H. Wolf (wolfj1@ohio.edu)

#### Department of Social Medicine, Ohio University Athens, OH, USA

**Introduction**

Despite ubiquitous public health campaigns urging mothers to breastfeed, breastfeeding rates were low in the United States during the late 19^th^ and early 20^th^ centuries [1]. Surveys conducted by visiting nurses in Chicago in 1908 indicate that only 39 percent of newborns were exclusively breastfed within a week of birth, a finding duplicated around the country. Mothers’ new habit of supplementing or wholly replacing human milk with cows’ milk crossed class lines: working-, as well as middle- and upper-class, women embraced these practices. The new custom was due, in part, to the work women performed, although the effect of work on infant feeding practice differed according to a woman’s class and the nature of her work.

**Findings**

Many upper-class women hired servants who took charge of all infant care, precluding breastfeeding. Working- and some middle-class women worked outside the home, as evidenced by the Little Mothers’ Clubs that appeared around the country as after school classes. Public health nurses conducted the clubs for the sole purpose of training schoolgirls to care for their tiny siblings when their mothers were at work. These nurses had long observed eight- and ten-year-old girls “carrying the baby and usually she knows more about the baby than her mother.” Beginning as an after school activity in 1912, and eventually made a permanent part of the urban school curriculum, the lessons offered by the clubs presented as their primary focus training in the preparation of cows’ milk for bottle-feeding. Of all working women, however, the most desperate were those who worked as wet nurses, who were invariably single women abandoned by their families or the father of their child. Without exception, the families who employed wet nurses forced them to place their own babies in foundling homes where, ironically, wet nurses’ infants were artificially fed. In an era before pasteurization and refrigeration, the vast majority of wet nurses’ infants died.

**Conclusion**

Breastfeeding was considered so vital in the late 19^th^ and early 20^th^ centuries that entire public health systems focused almost solely on the consequences of artificial feeding, attempting to convince mothers of the value of breastfeeding and the dangers of cows’ milk. Yet paradoxically, in the class-based society of the early 20^th^ century urban United States, breastfeeding was treated as an individual value rather than a shared societal value and thus was valued and maintained for some children and not for others.

**References**

1. Jacqueline H. Wolf, *Don’t Kill Your Baby: Public Health and the Decline of Breastfeeding in the 19th and 20th Centuries.* Columbus, OH: Ohio State University Press, 2001, 9-73.

